# *The Plant Genome* special issue: Advances in genomic selection and application of machine learning in genomic prediction for crop improvement

**DOI:** 10.1002/tpg2.20178

**Published:** 2021-11-22

**Authors:** Rajeev K. Varshney

**Affiliations:** ^1^ Center of Excellence in Genomics & Systems Biology International Crops Research Institute for the Semi‐Arid Tropics (ICRISAT) Hyderabad India; ^2^ State Agricultural Biotechnology Centre, Centre for Crop and Food Innovation, Food Futures Institute Murdoch University Murdoch Western Australia Australia

Since Meuwissen et al. (2001) proposed the idea of using genome‐wide marker information for prediction of the genetic worth of untested individuals, the concept of genomic selection (GS) has spawned a series of publications over the last two decades in animals and plants alike. Advances in genomic profiling and phenotyping have given a strong impetus to application of GS in plant breeding (Crossa et al., 2017; Varshney et al., 2017). Empirical and simulation studies suggest that GS can improve the rate of genetic gain in plant breeding programs via influencing the various parameters of the breeder's equation (Sinha et al., 2021). Concurrent advances in methods of genomic prediction including parametric and non‐parametric have resulted in considerable improvements in the prediction accuracies of different GS models. The rising complexity of datasets emanating from high throughput genotyping, phenotyping and omics systems calls for harnessing the enormous potential of machine learning (ML) tools for prediction of plant performance (Varshney et al., 2021a). This issue on “Advances in genomic selection and application of machine learning in genomic prediction” presents 14 articles from leading experts in this field. Key highlights of these articles are summarized here in this editorial.

In the first article, Sandhu et al. (2021) demonstrated the utility of machine‐ and deep learning models for prediction of grain yield and grain protein content in wheat based on six different spectral reflectance indices. Enhanced prediction accuracies were obtained with machine‐ and deep learning models *viz*. Random forest and multilayer perceptron as compared with the conventional genomic prediction models including genomic best linear unbiased predictor and Bayesian models. The study also established the superiority of multi‐trait GS models over uni‐trait GS models. Extending genomic prediction approach to count data, Montesinos‐López et al. (2021a) applied Poisson deep neural network (PDNN) and obtained better prediction performance with PDNN compared with Bayesian regression and generalized Poisson regression methods. The advantage of the PDNN was evident in the case of count data on large datasets or for datasets with fewer observations than independent variables.

Machine learning is expected to play an important role in GS (Varshney et al., 2021b, 2021a). In this issue, Bayer et al. (2021) presented perspectives on using ML in GS with a focus on addressing nonadditive effects in GS models. The review also provides researchers with potential solutions to the challenges of applying ML approaches to GS including the difficulties encountered in interpretability of ML and parameter optimization. The authors also highlight the significance of gene content information from pangenome data in leveraging the training process in genomic prediction. In another article, Montesinos‐López et al. (2021b) discussed that the ability of ML methods to capture complex patterns and large datasets renders these highly relevant to GS. The authors underscore key future researchable areas that will help adapt DL to GS strategies, thus accelerating the decision making process in plant breeding.

Genomic prediction has emerged as a promising alternative to the conventional approach of extensive field evaluations for the development of heterotic groups in several crop species (Bohra et al., 2016). In pigeonpea, Saxena et al. (2021) trained a GS model on a population of 396 lines and 435 hybrids to predict the performance of all 78,210 possible single‐cross hybrids based on 396 lines. The genomic prediction approach proposed in this article paves the way for establishment of heterotic groups and identification of high‐yielding heterotic patterns in different crops, which in turn will be crucial for ascertaining long‐term gain in hybrid breeding.

Given the higher efficiency of index selection over tandem selection or independent culling levels, Sweeney et al. (2021) provided empirical evidence in support of GS based on selection indices targeting multiple traits in two‐row spring malting barley. The superior performance of GS over phenotypic selection was demonstrated for selection index value, height, and pre‐harvest sprouting resistance. Furthermore, the study reinforced the significance of phenotyping since phenotyping errors contributed to low realized gain for spot blotch. This study suggested that GS might be particularly useful in case of incomplete phenotyping scenarios. GS causes an increase in inbreeding but controlled mating strategy and optimum contribution selection (OCS) may help reduce inbreeding levels in GS schemes (Varshney et al., 2021c). Genomic selection with OCS improved grain yield, Fe and Zn besides reducing time to cooking in African common bean (Saradadevi et al., 2021). The five cycles of the GS strategy proposed in this article could potentially lead to the development of the biofortified beans with enhanced Fe content (22 mg kg^−1^ more than the local check). In fact, GS with OCS with crop wild relatives has also been suggested an effective pre‐breeding approach for crop improvement (Bohra et al. 2021).

Empirical evidence in support of the supremacy of ML methods for genomic prediction over simple linear methods is currently limited. By obscuring the access of network to marker data, Ubbens et al. (2021) showed that the ML methods consider genetic relatedness instead of the marker effects, possibly explaining the gap in expected and realized potential of ML in genomic prediction. The study proposed to avoid shortcut learning in genomic data for enhancing the performance of ML methods for genomic prediction.

In another study, Islam et al. (2021) applied parametric and non‐parametric models in sugarcane for predicting resistance against rust disease. The higher prediction ability of non‐parametric GS models could be ascribed to the prevalence of non‐additive effects in disease resistance. Notably, inclusion of known rust resistance gene *Bru1* gene as a fixed effect substantially enhanced the performance of the parametric model, particularly in the case of fewer number of DNA markers and small training population size.

Strong interaction with environment complicates the selection for quantitative traits in plant breeding programs. Incorporation of genotype × environment interaction (GE) in GS models has considerably improved prediction abilities. Crespo‐Herrera et al. (2021) compared the performance of different models in the case of sparse testing in wheat and found that the GS model with GE incorporated had better accuracies than other models that did not account for this interaction. Inclusion of GE in GS models could prove immensely useful for optimum allocation of resources in plant breeding programs, such as the number of overlapping lines for multi‐environment field testing. Lell et al. (2021) applied a resampling approach on the large‐scale published genotyping and phenotyping data of 1,604 winter wheat hybrids for testing various biometric models to optimize the design of multi‐environment yield trials. Balanced environmental sampling caused an increase in prediction accuracy.

Merrick and Carter (2021) showed the utility of the GS approach for selecting plant traits with unknown complex genetic architecture that are substantially influenced by the environment. By comparing different GS models for deep‐sown seedling emergence in wheat, the authors recorded slightly higher performance of non‐parametric models in the case of phenotyping data combined over the years. No significant difference between parametric and non‐parametric models within individual years suggested little need to account for non‐additive effects in such scenarios. The choice of models relied heavily on the structure of the training population.

Genomic selection eases selection of plant traits such as end‐use quality, which are evaluated at the final stage, and their evaluation demands considerable investments in terms of time and cost. For end‐use quality traits in wheat, Zhang‐Biehn et al. (2021) showed that the inclusion of indirect selection in GS models improved prediction accuracies. Importantly, GS approaches harnessing secondary traits as covariates or correlated response variables create novel possibilities for selecting breeding lines with high levels of both quality and yield traits, which are generally known to show negative correlations. The authors also performed GWAS to analyze mixograph mixing time and bake mixing time, two end‐use quality traits in winter wheat; and reported SNP markers for future marker‐assisted selection.

A remarkable decline in the cost of genotyping has fueled genomic characterization of large collection of germplasms archived in genebanks. The use of genotyping and phenotyping data on germplasm collections to predict the genetic worth of the untested germplasm has been referred to as turbocharging genebanks. Dzievit et al. (2021) trained models from the Maize Association Population (MAP) to obtain genomic‐estimated breeding values (GEBVs) for individuals from the Ames Diversity Panel. Combining GEBVs and an upper bound for reliability (U‐value) for selecting germplasms from genebanks will accelerate trait improvement while retaining the genetic diversity in the breeding program.

In recent years, selection of lines is shifting from a few loci to whole‐genome sequencing and/or genome‐wide profiling data in crop breeding programs. Such efforts are not only in industrial and major crops like maize, soybean, wheat and rice but also in minor or so‐called orphan crops like chickpea and cassava. Going forward, GS and ML application in genomic prediction are going to be routine in plant breeding in the future. ML and artificial intelligence approaches are expected for making the best use of the massive amount of high‐density sequencing/genomics and phenotyping data. This will allow for better prediction of lines and their crosses and enhance the precision and efficiency of plant breeding in coming years. The GS along with other fast‐forward breeding approaches are expected to develop climate resilient and better nutrition varieties in a faster manner (Varshney et al., 2021a). Furthermore, it is also essential to establish and deploy rapid delivery systems to harness the full potential of genetics and plant breeding to contribute to feeding 10 billion people by 2050 (Varshney et al., 2021b).
